# Supplementary material for: Potent and Non-Cytotoxic Antibacterial Compounds against Methicillin-Resistant Staphylococcus aureus Isolated from Psiloxylon mauritianum, A Medicinal Plant from Reunion Island
Source: Molecules. 2020 Aug 5;25(16):3565. doi: 10.3390/molecules25163565 (PMC7465348; doi:10.3390/molecules25163565)
Supplement: Supplementary file 1 [file molecules-25-03565-s001.pdf]

SUPPORTING MATERIALS FOR

Communication

Potent and Non-Cytotoxic Antibacterial Compounds Against Methicillin-Resistant *Staphylococcus aureus* Isolated from *Psiloxylon mauritianum,* A Medicinal Plant from Reunion Island

Jonathan Sorres [1,2,*], Amandine André [2,4], Elsa Van Elslande [2], Didier Stien [3] and Véronique Eparvier [2,*]

[1] Association DESIBER, 98 rue Roger Payet, Rivière des Pluies, La Réunion, 97438 Sainte Marie, France
[2] CNRS, Institute of Chemistry of Natural Substances UPR2301, University of Paris-Saclay, 91198 Gif-sur-Yvette, France; andramandine@gmail.com (A.A.); elsa.van-elslande@cnrs.fr (E.V.E.)
[3] Laboratory of Biodiversity and Microbial Biotechnologies (LBBM), Sorbonne University, CNRS, 75006 Paris, France, UPMC Univ Paris 06, Banyuls-sur-Mer oceanological observatory, 66650 Banyuls-sur-Mer, France; didier.stien@cnrs.fr
[4] Laboratoire Shigeta, 62 boulevard Davout, 75020 Paris, France
* Correspondence: jon.sorres@gmail.com (J.S.); veronique.eparvier@cnrs.fr (V.E.); Tel.: +33-169-823-679 (V.E.)

1. Table of Figures

Figure S1: ^{1}H NMR (500 MHz, $(CD_3)_2CO$ spectrum of aspidin BB (**1**)
Figure S2: ^{13}C NMR (125 MHz, $(CD_3)_2CO$ spectrum of aspidin BB (**1**)
Figure S3. COSY NMR (500 MHz, $(CD_3)_2CO$ spectrum of aspidin BB (**1**)
Figure S4. HSQC NMR (500 MHz, $(CD_3)_2CO$ spectrum of aspidin BB (**1**)
Figure S5. HMBC NMR (500 MHz, $(CD_3)_2CO$ spectrum of aspidin BB (**1**)
Figure S6. ^{1}H NMR (500 MHz, $(CD_3)_2CO$ spectrum of aspidin VB (**2**)
Figure S7. ^{13}C NMR (125 MHz, $(CD_3)_2CO$ spectrum of aspidin VB (**2**)
Figure S8. COSY NMR (500 MHz, $(CD_3)_2CO$ spectrum of aspidin VB (**2**)
Figure S9. HSQC NMR (500 MHz, $(CD_3)_2CO$ spectrum of aspidin VB (**2**)
Figure S10. HSQC NMR (500 MHz, $(CD_3)_2CO$ spectrum of aspidin VB (**2**)
Figure S11. ROESY NMR (500 MHz, $(CD_3)_2CO$ spectrum of aspidin VB (**2**)
Figure S12. ^{1}H NMR (700 MHz, $(CD_3)_2CO$ spectrum of the ursolic acid (**3**):oleanic acid (**4**) (6:4) mixture
Figure S13. ^{13}C NMR (175 MHz, $(CD_3)_2CO$ spectrum of the ursolic acid:oleanic acid (6:4) mixture
Figure S14. Predicted molecular properties of aspidin BB (**1**) obtained with SwissADME
Figure S15. Predicted molecular properties of aspidin VB (**2**) obtained with SwissADME

1. List of Tables

Table S1. X-ray crystal data and structure refinement of aspidin BB **(1).**
Table S2. Fractional Atomic Coordinates (×104) and Equivalent Isotropic Displacement Parameters (Å2×103) for aspidin BB **(1).**
Table S3. Anisotropic Displacement Parameters (Å2×103) for aspidin BB **(1).**
Table S4. Bond Lengths for aspidin BB **(1).**
Table S5. Bond Angles for aspidin BB **(1).**
Table S6. Torsion Angles for aspidin BB **(1).**
Table S7. Hydrogen Atom Coordinates (Å×104) and Isotropic Displacement Parameters (Å2×103) for aspidin BB **(1).**
Table S8. Atomic Occupancy for aspidin BB **(1).**

Table S9. X-ray crystal data and structure refinement of aspidin VB **(2)**.
Table S10. Fractional Atomic Coordinates (×10^4) and Equivalent Isotropic Displacement Parameters (Å^2×10^3) for aspidin VB **(2)**.
Table S11. Anisotropic Displacement Parameters (Å^2×103) for aspidin VB **(2)**.
Table S12. Bond Lengths for aspidin VB **(2)**.
Table S13. Bond Angles for aspidin VB **(2)**.
Table S14. Torsion Angles for aspidin VB **(2)**.
Table S15. Hydrogen Atom Coordinates (Å×10^4) and Isotropic Displacement Parameters (Å^2×10^3) for aspidin VB **(2)**.
Table S16. Atomic Occupancy for aspidin VB **(2)**.

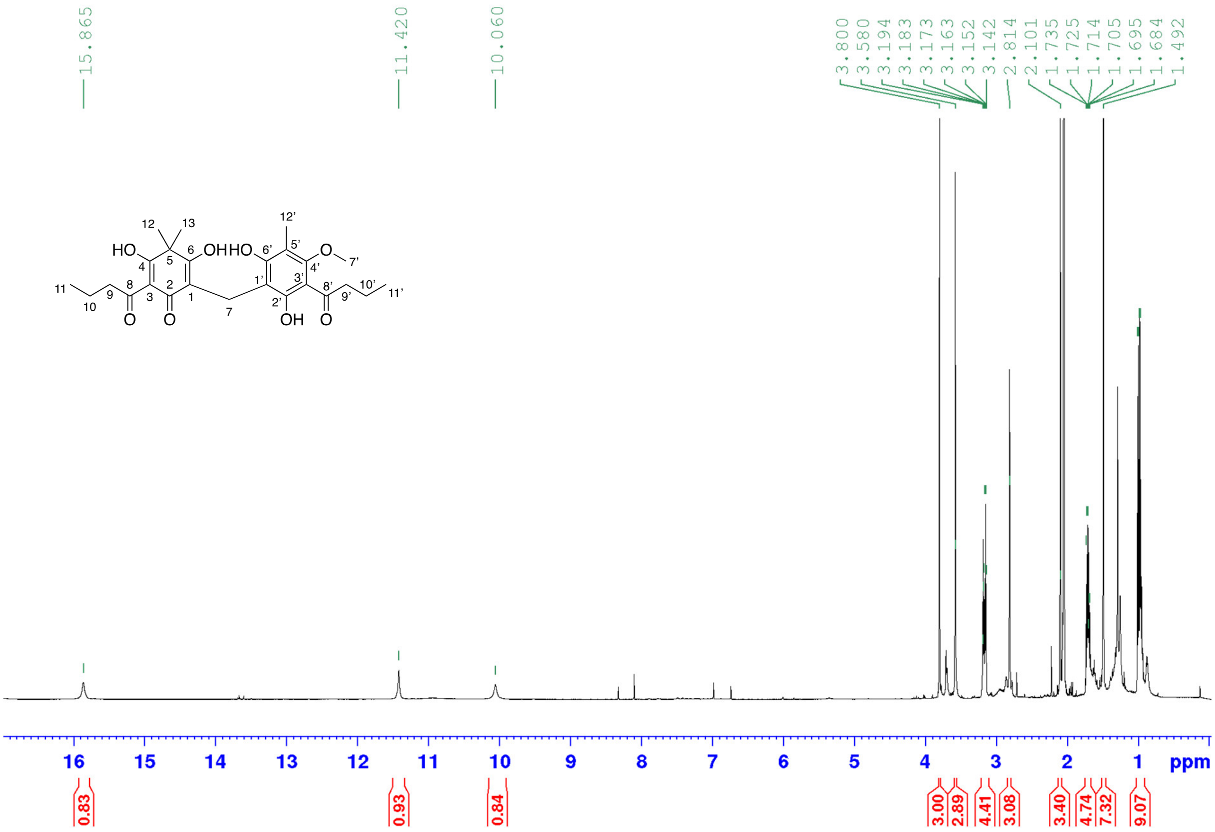

Figure 1. ^{1}H NMR (500 MHz, $(CD_3)_2CO$) spectrum of Aspidin BB **1.**

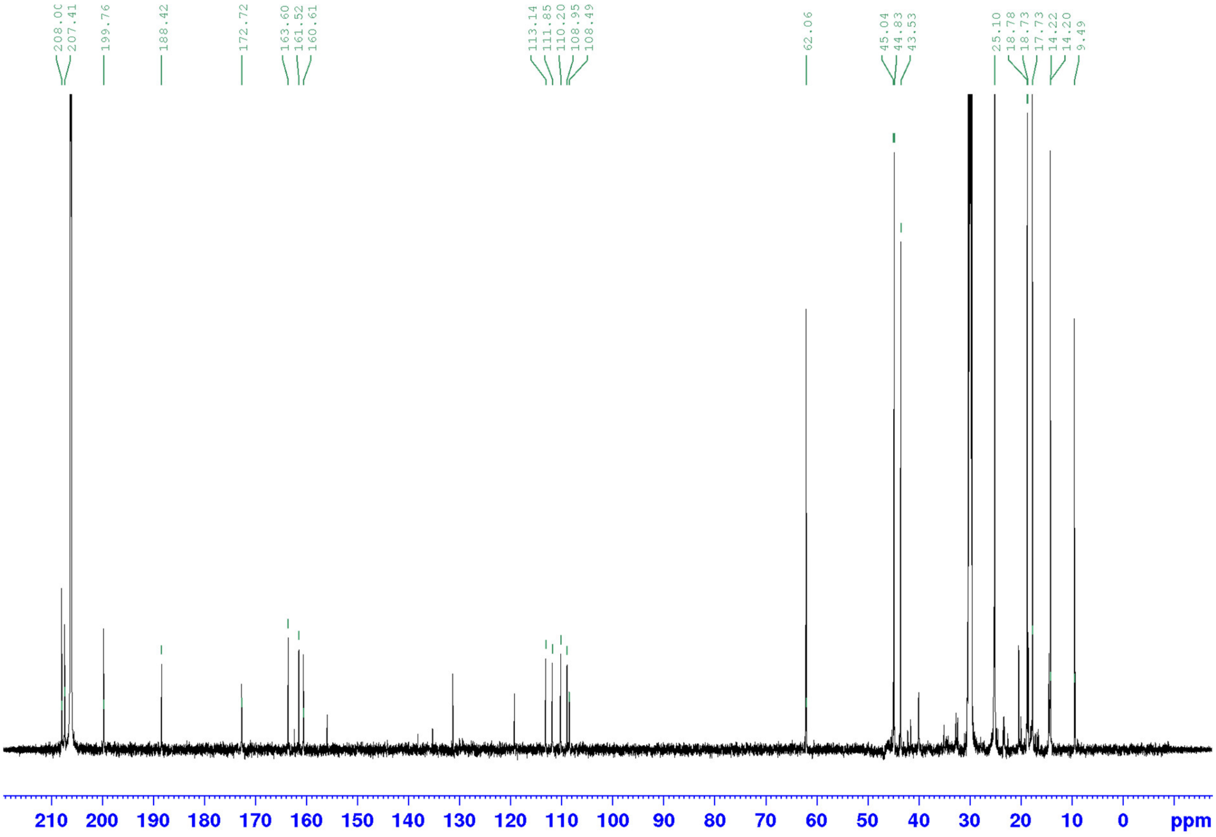

Figure 2. ^{13}C NMR (125 MHz, $(CD_3)_2CO$) spectrum of aspidin BB **1.**

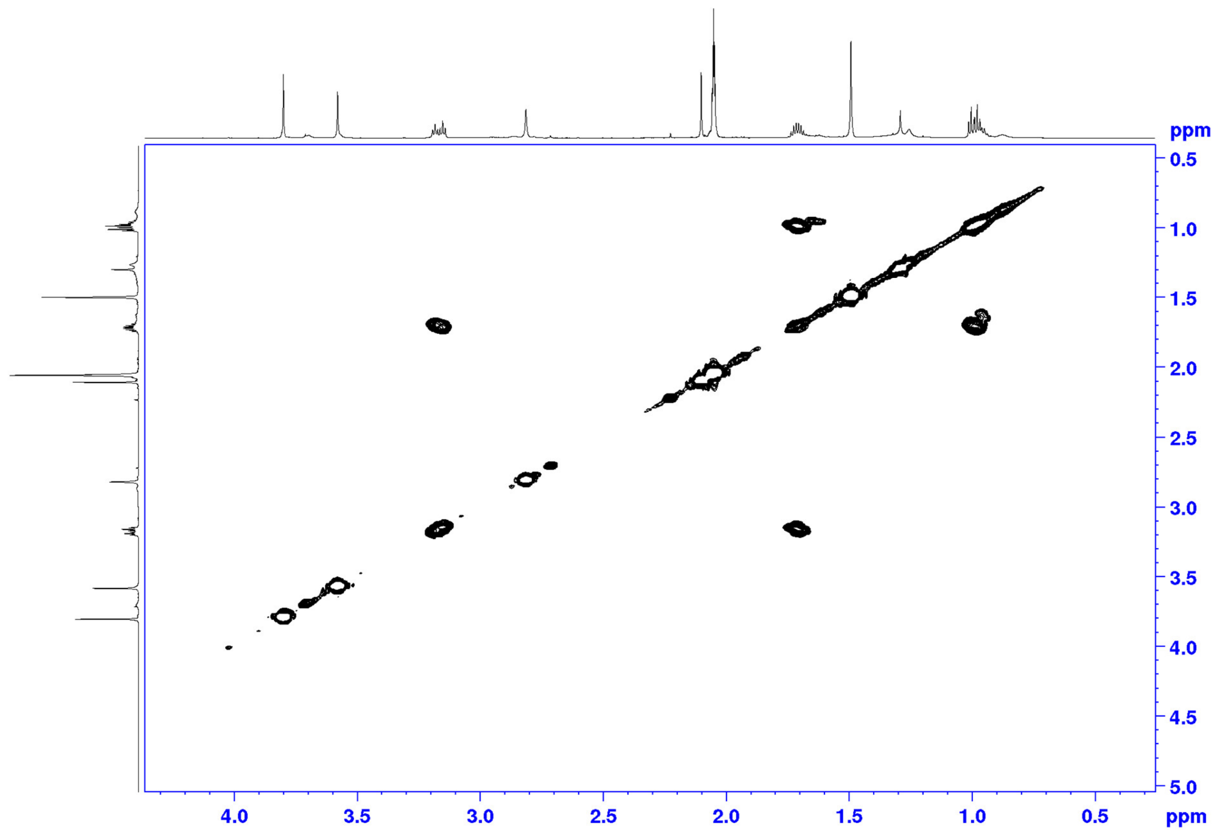

Figure 3. COSY NMR (500 MHz, $(CD_3)_2CO$) spectrum of aspidin BB **1.**

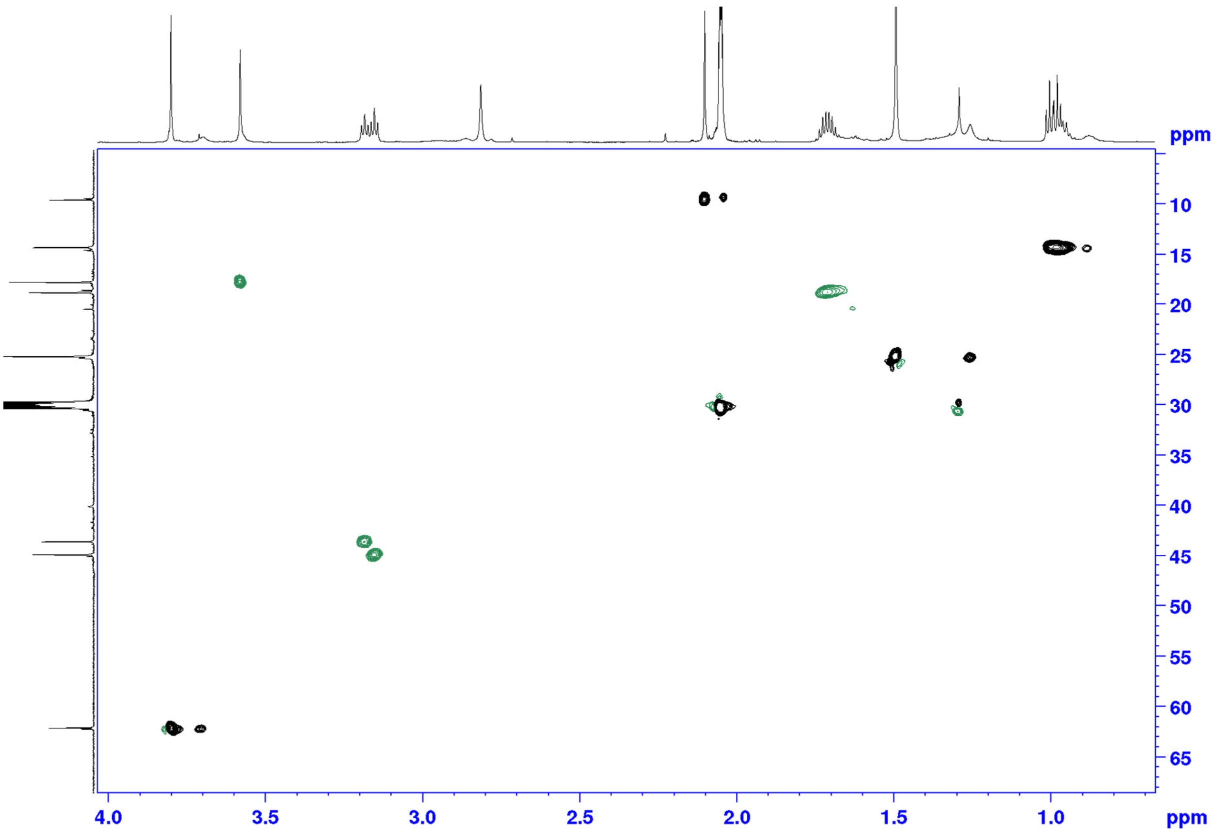

Figure 4. HSQC NMR (500 MHz, $(CD_3)_2CO$) spectrum of aspidin BB **1.**

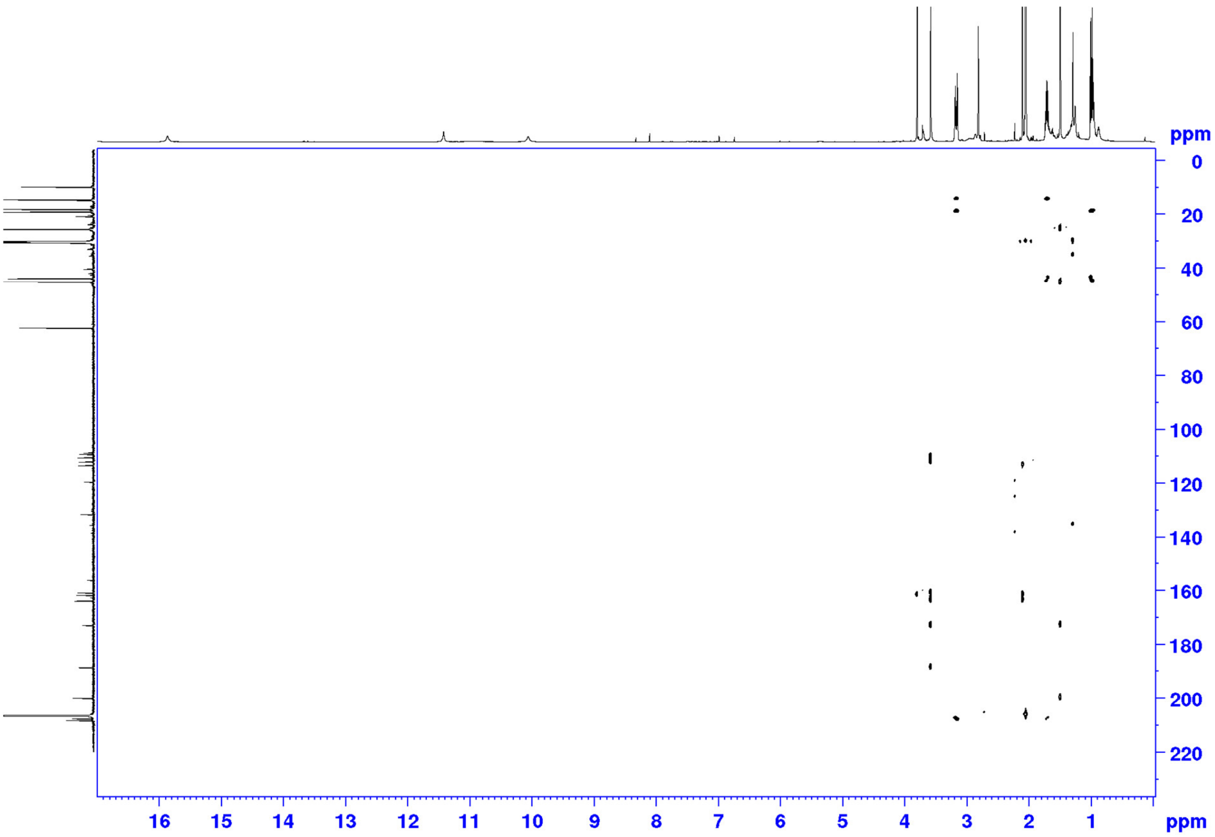

Figure 5. HMBC NMR (500 MHz, $(CD_3)_2CO$) spectrum of aspidin BB **1.**

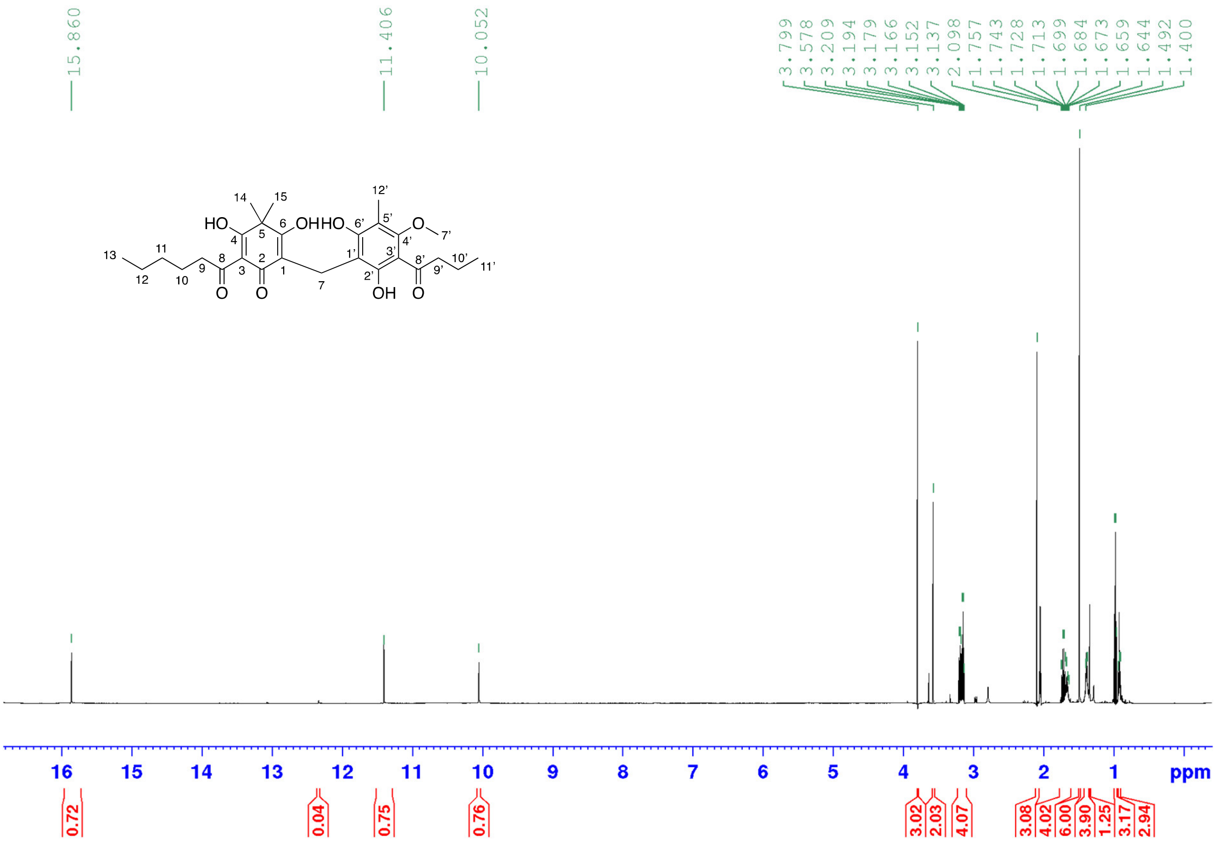

Figure 6. ^{1}H NMR (500 MHz, $(CD_3)_2CO$) spectrum of aspidin VB **2.**

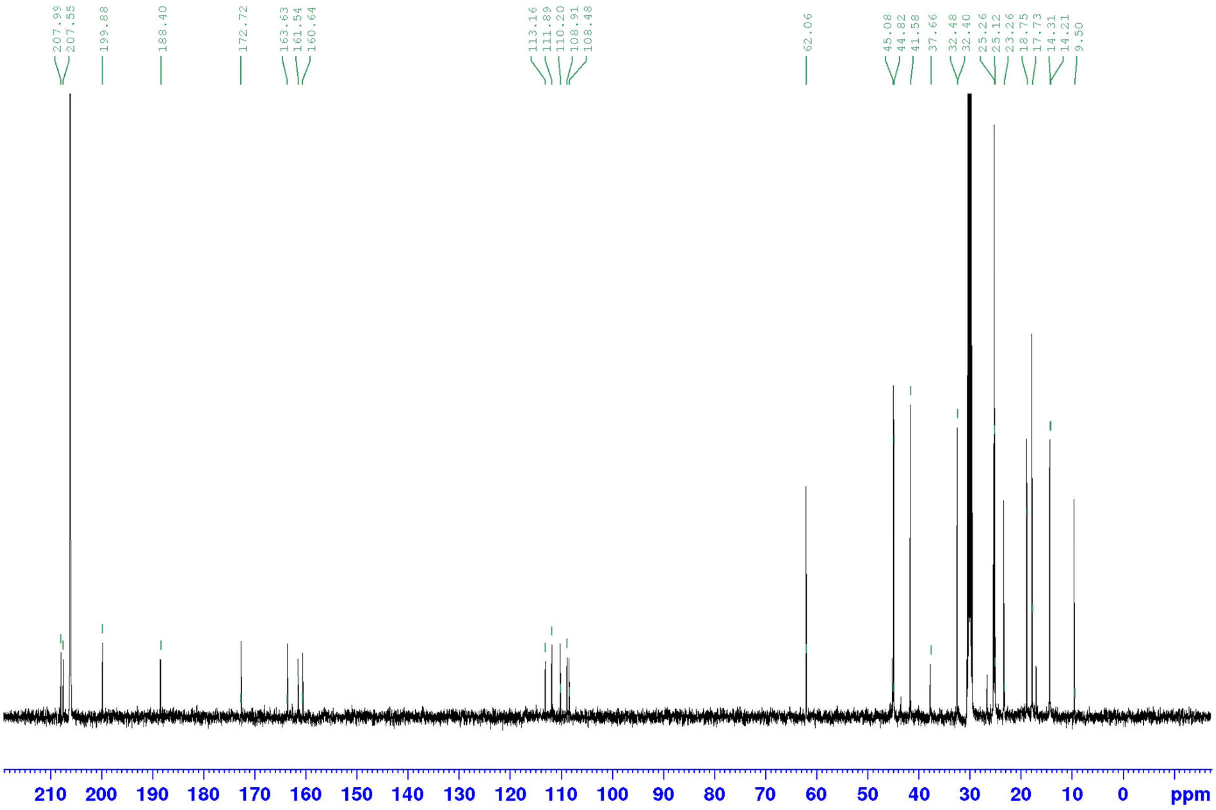

Figure 7. ^{13}C NMR (125 MHz, $(CD_3)_2CO$) spectrum of aspidin VB **2.**

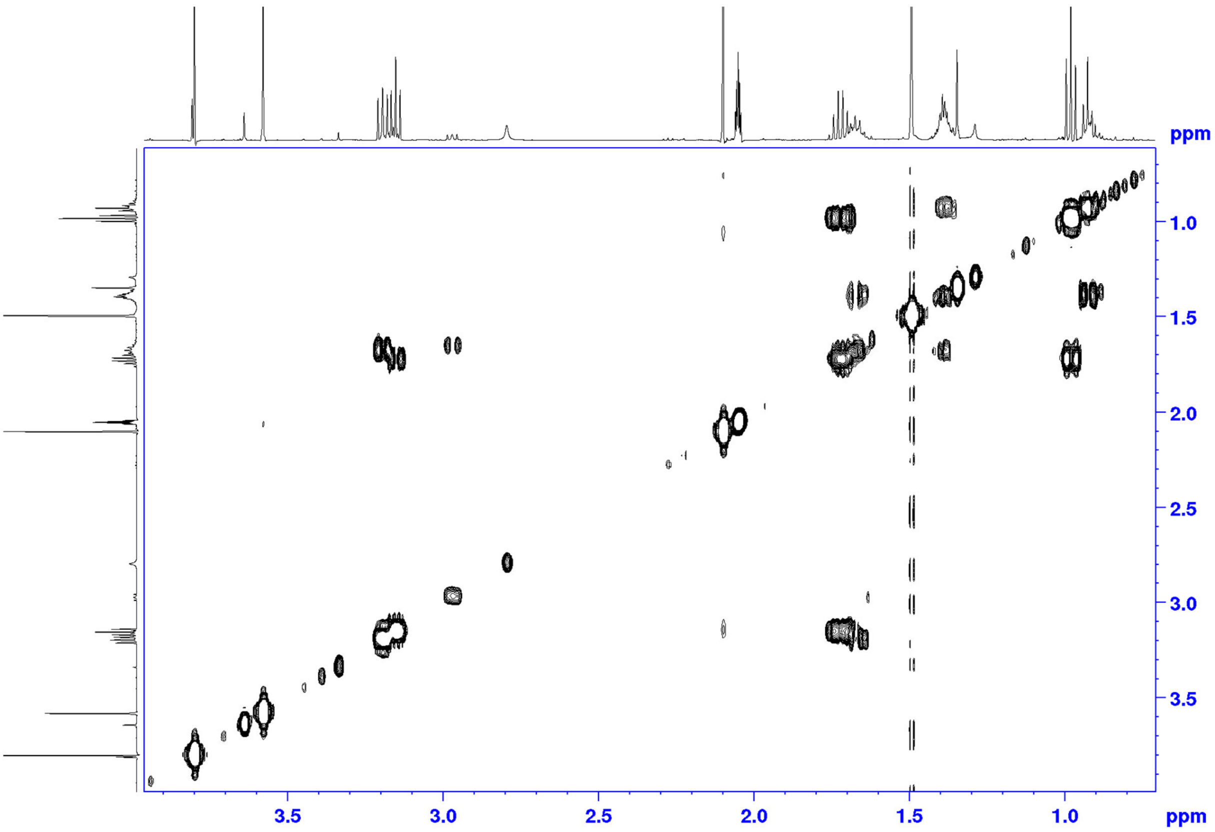

Figure 8. COSY NMR (500 MHz, $(CD_3)_2CO$) spectrum of aspidin VB **2.**

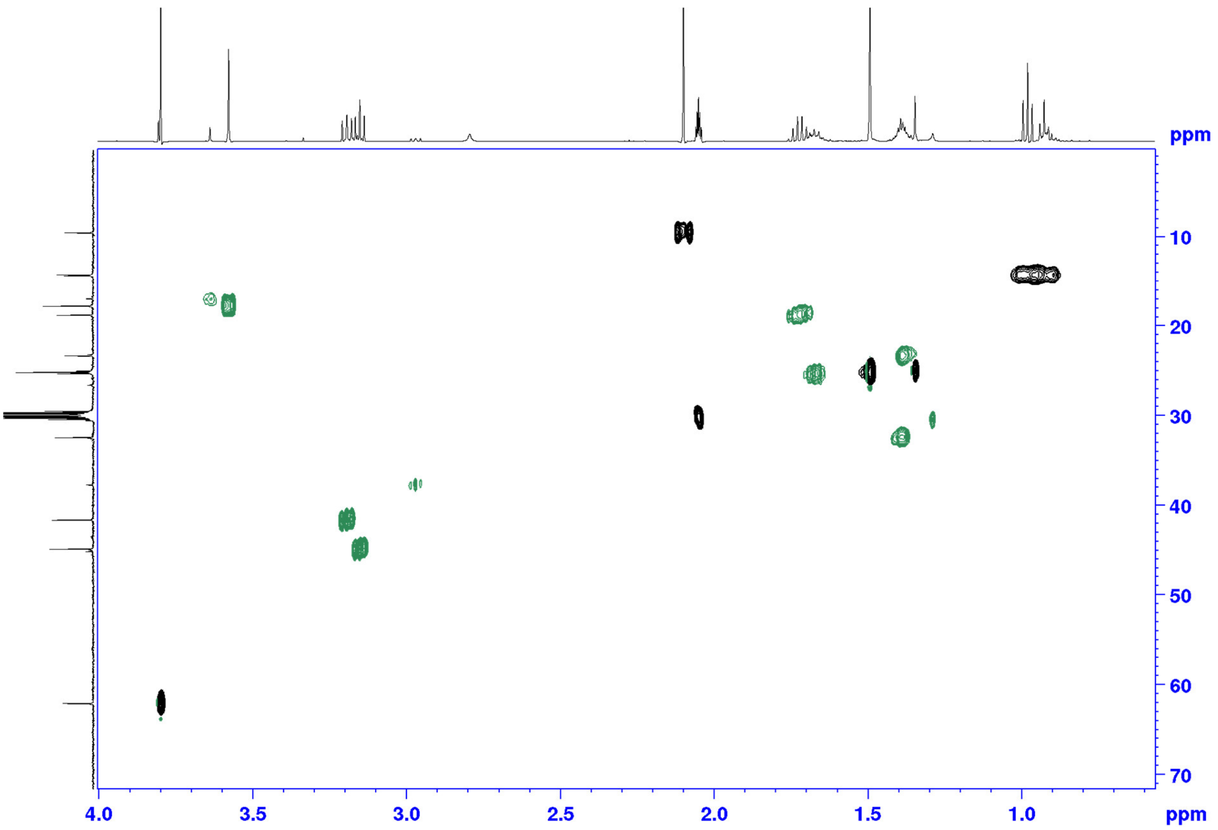

Figure 9. HSQC NMR (500 MHz, $(CD_3)_2CO$) spectrum of aspidin VB **2.**

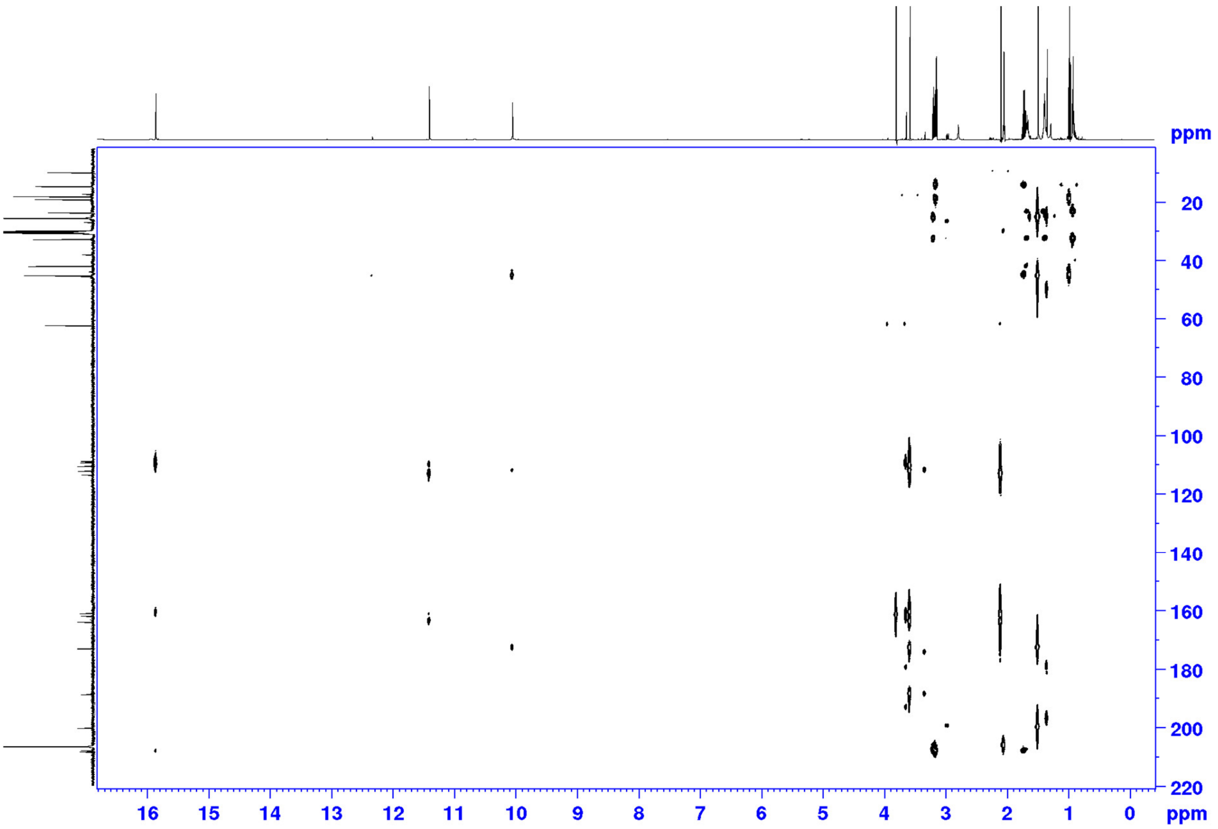

Figure 10. HSQC NMR (500 MHz, $(CD_3)_2CO$) spectrum of aspidin VB **2.**

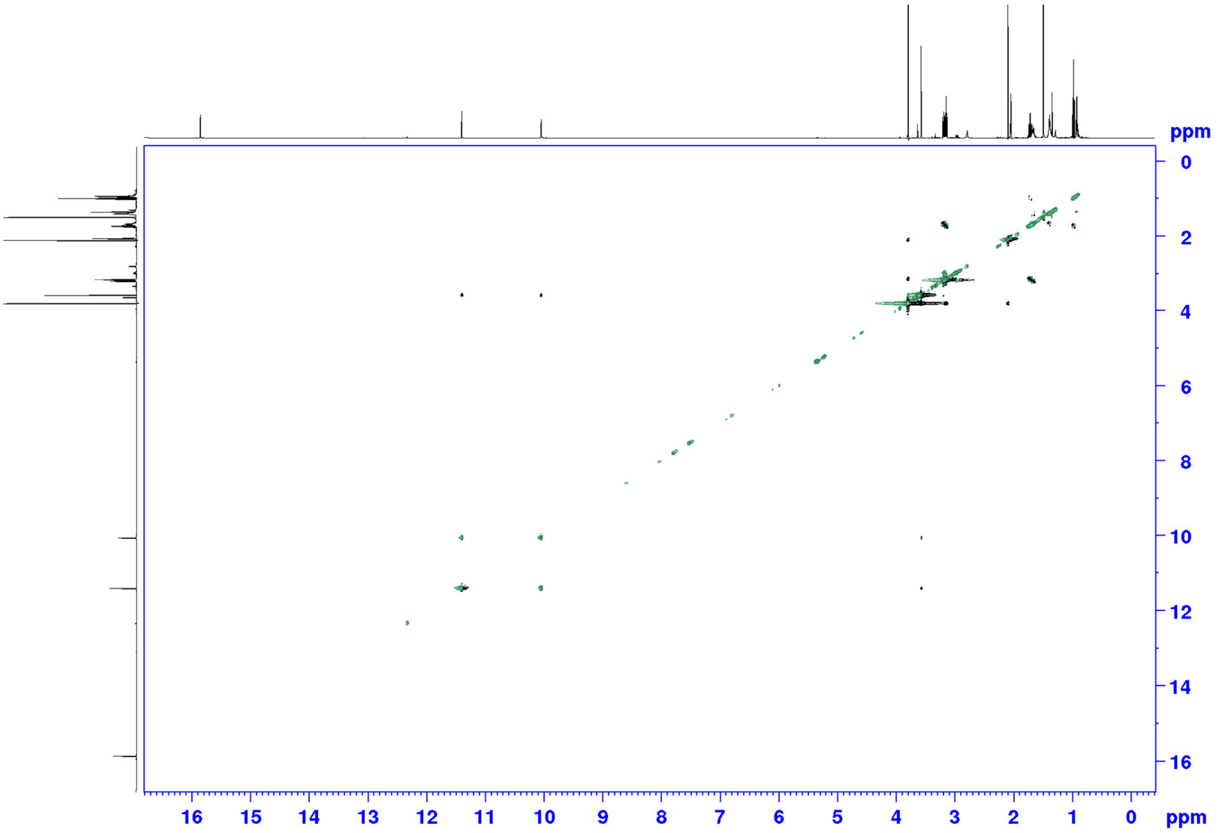

Figure 11. ROESY NMR (500 MHz, $(CD_3)_2CO$) spectrum of aspidin VB **2.**

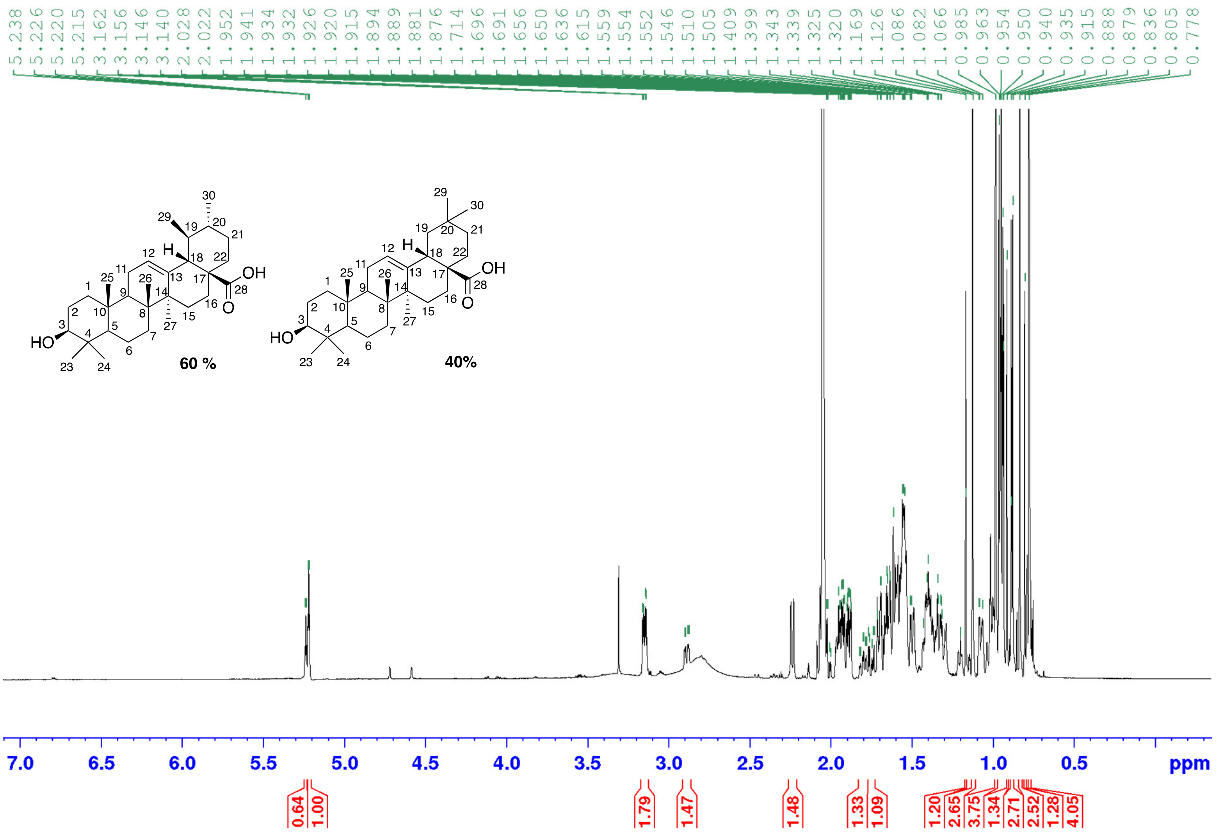

Figure 12. ^{1}H NMR (700 MHz, $(CD_3)_2CO$) spectrum of the ursolic acid (**3**):oleanic acid (**4**) (6:4) mixture.

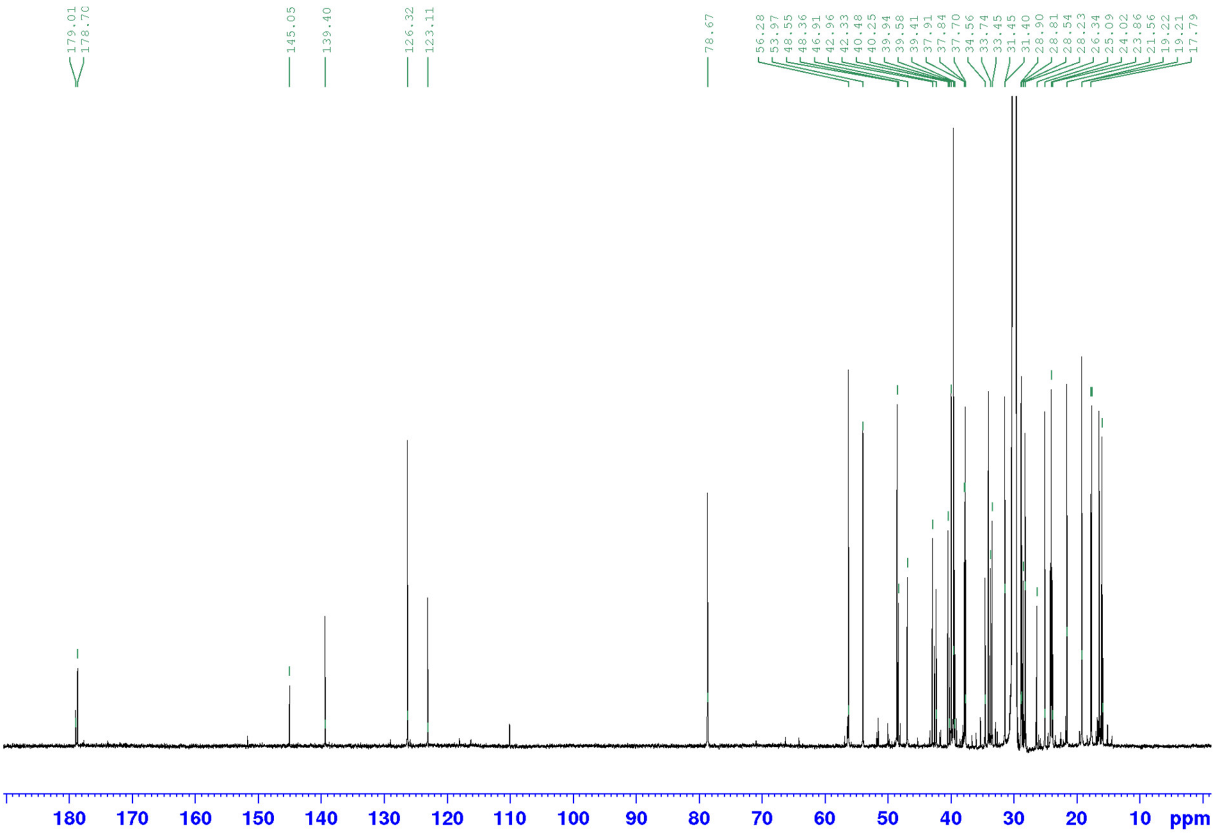

Figure 13. ^{13}C NMR (175 MHz, $(CD_3)_2CO$) spectrum of the ursolic acid:oleanic acid (6:4) mixture.

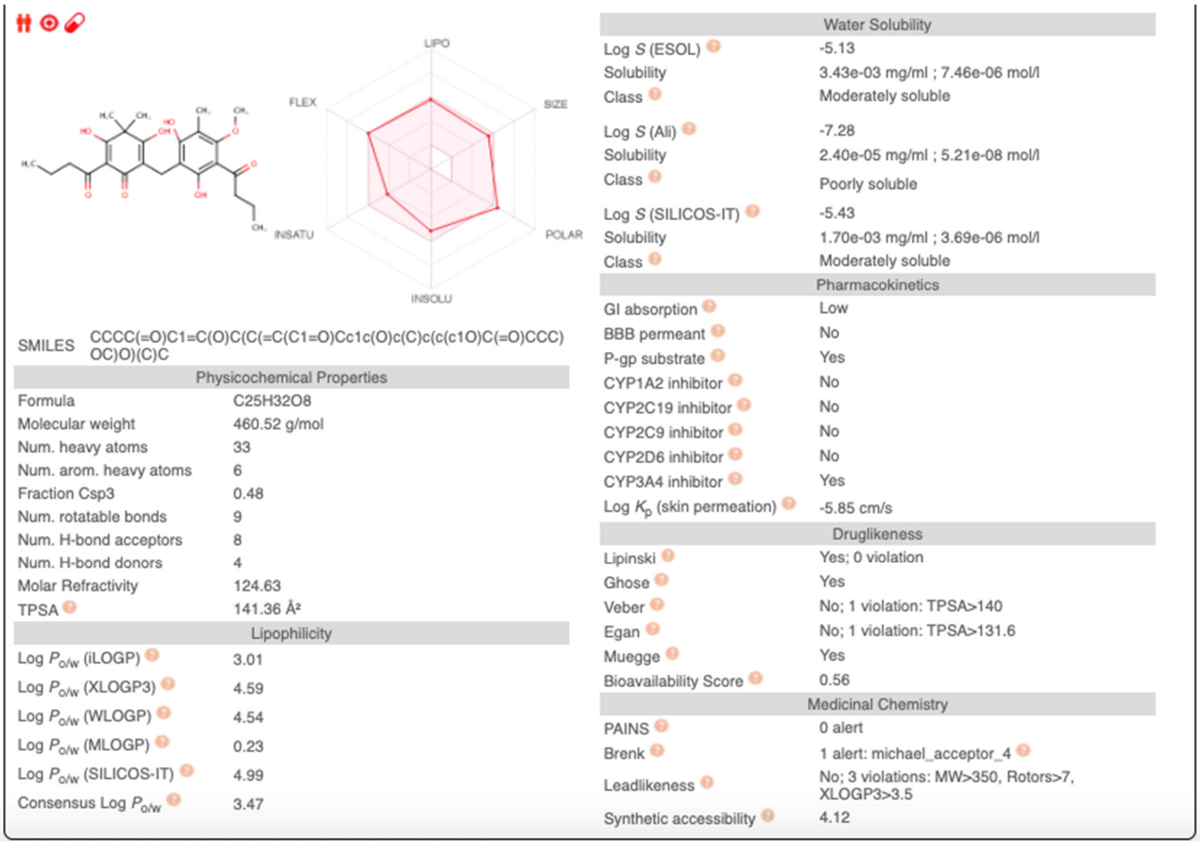

SMILES CCCC(=O)C1=C(O)C(C(=C(C1=O)Cc1c(O)c(C)c(c(c1O)C(=O)CCC)OC)O)(C)C

Physicochemical Properties	
Formula	C25H32O8
Molecular weight	460.52 g/mol
Num. heavy atoms	33
Num. arom. heavy atoms	6
Fraction Csp3	0.48
Num. rotatable bonds	9
Num. H-bond acceptors	8
Num. H-bond donors	4
Molar Refractivity	124.63
TPSA	141.36 Å²

Lipophilicity	
Log $P_{o/w}$ (iLOGP)	3.01
Log $P_{o/w}$ (XLOGP3)	4.59
Log $P_{o/w}$ (WLOGP)	4.54
Log $P_{o/w}$ (MLOGP)	0.23
Log $P_{o/w}$ (SILICOS-IT)	4.99
Consensus Log $P_{o/w}$	3.47

Water Solubility	
Log *S* (ESOL)	-5.13
Solubility	3.43e-03 mg/ml ; 7.46e-06 mol/l
Class	Moderately soluble
Log *S* (Ali)	-7.28
Solubility	2.40e-05 mg/ml ; 5.21e-08 mol/l
Class	Poorly soluble
Log *S* (SILICOS-IT)	-5.43
Solubility	1.70e-03 mg/ml ; 3.69e-06 mol/l
Class	Moderately soluble

Pharmacokinetics	
GI absorption	Low
BBB permeant	No
P-gp substrate	Yes
CYP1A2 inhibitor	No
CYP2C19 inhibitor	No
CYP2C9 inhibitor	No
CYP2D6 inhibitor	No
CYP3A4 inhibitor	Yes
Log K_p (skin permeation)	-5.85 cm/s

Druglikeness	
Lipinski	Yes; 0 violation
Ghose	Yes
Veber	No; 1 violation: TPSA>140
Egan	No; 1 violation: TPSA>131.6
Muegge	Yes
Bioavailability Score	0.56

Medicinal Chemistry	
PAINS	0 alert
Brenk	1 alert: michael_acceptor_4
Leadlikeness	No; 3 violations: MW>350, Rotors>7, XLOGP3>3.5
Synthetic accessibility	4.12

Figure 14. Predicted molecular properties of aspidin BB (**1**) obtained with SwissADME.

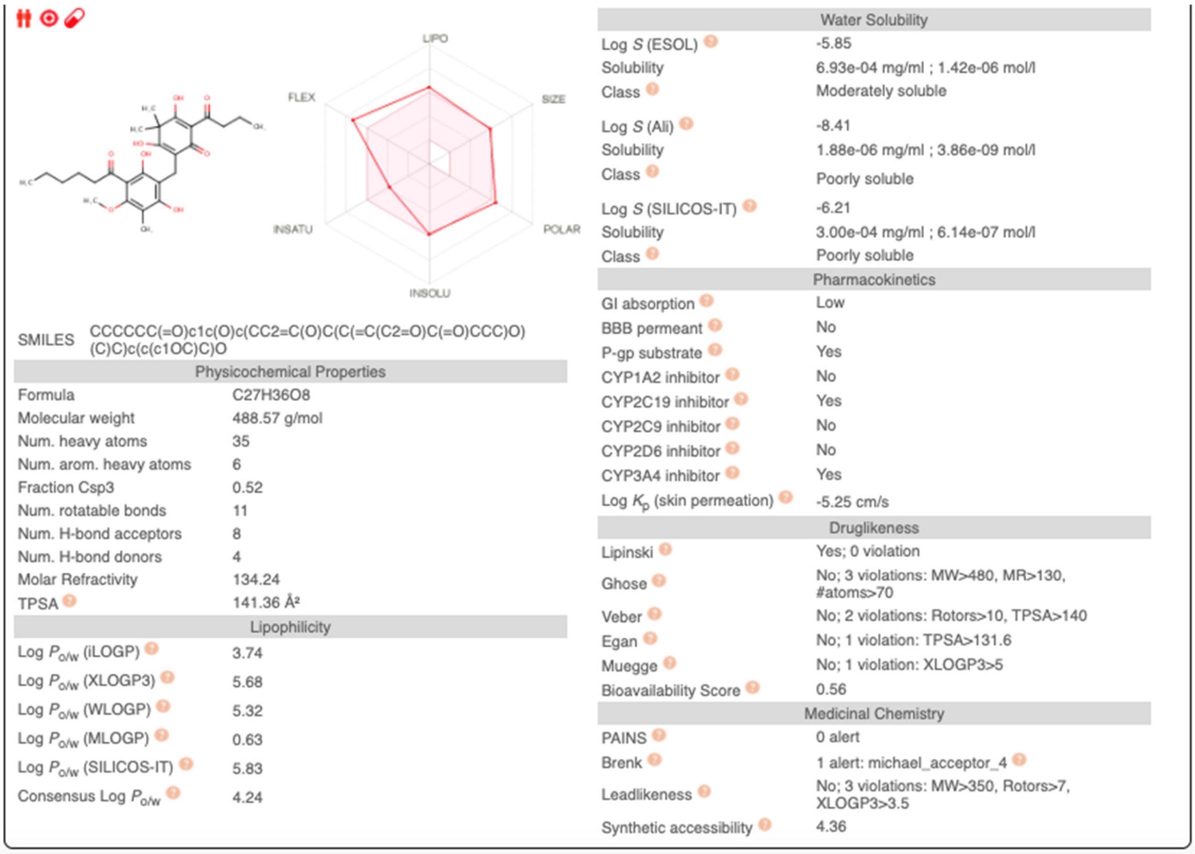

SMILES CCCCCC(=O)c1c(O)c(CC2=C(O)C(C(=C(C2=O)C(=O)CCC)O)(C)C)c(c(c1OC)C)O

Physicochemical Properties	
Formula	C27H36O8
Molecular weight	488.57 g/mol
Num. heavy atoms	35
Num. arom. heavy atoms	6
Fraction Csp3	0.52
Num. rotatable bonds	11
Num. H-bond acceptors	8
Num. H-bond donors	4
Molar Refractivity	134.24
TPSA	141.36 Å²

Lipophilicity	
Log $P_{o/w}$ (iLOGP)	3.74
Log $P_{o/w}$ (XLOGP3)	5.68
Log $P_{o/w}$ (WLOGP)	5.32
Log $P_{o/w}$ (MLOGP)	0.63
Log $P_{o/w}$ (SILICOS-IT)	5.83
Consensus Log $P_{o/w}$	4.24

Water Solubility	
Log *S* (ESOL)	-5.85
Solubility	6.93e-04 mg/ml ; 1.42e-06 mol/l
Class	Moderately soluble
Log *S* (Ali)	-8.41
Solubility	1.88e-06 mg/ml ; 3.86e-09 mol/l
Class	Poorly soluble
Log *S* (SILICOS-IT)	-6.21
Solubility	3.00e-04 mg/ml ; 6.14e-07 mol/l
Class	Poorly soluble

Pharmacokinetics	
GI absorption	Low
BBB permeant	No
P-gp substrate	Yes
CYP1A2 inhibitor	No
CYP2C19 inhibitor	Yes
CYP2C9 inhibitor	No
CYP2D6 inhibitor	No
CYP3A4 inhibitor	Yes
Log K_p (skin permeation)	-5.25 cm/s

Druglikeness	
Lipinski	Yes; 0 violation
Ghose	No; 3 violations: MW>480, MR>130, #atoms>70
Veber	No; 2 violations: Rotors>10, TPSA>140
Egan	No; 1 violation: TPSA>131.6
Muegge	No; 1 violation: XLOGP3>5
Bioavailability Score	0.56

Medicinal Chemistry	
PAINS	0 alert
Brenk	1 alert: michael_acceptor_4
Leadlikeness	No; 3 violations: MW>350, Rotors>7, XLOGP3>3.5
Synthetic accessibility	4.36

Figure 15. Predicted molecular properties of aspidin VB (**2**) obtained with SwissADME.

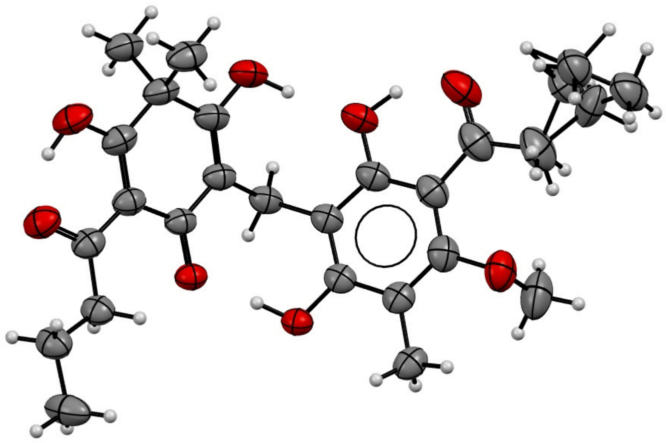

Table 1. X-ray crystal data and structure refinement of aspidin BB (**1**).

**Identification code**	**compound_1**
Empirical formula	$C_{25}H_{32}O_8$
Formula weight	460.50
Temperature/K	100.15
Crystal system	orthorhombic
Space group	Pbca
a/Å	9.4450(10)
b/Å	17.0100(10)
c/Å	28.7680(15)
α/°	90
β/°	90
γ/°	90
Volume/Å^3	4621.9(6)
Z	8
ρ_{calc}g/cm^3	1.324
μ/mm^{-1}	0.104
F(000)	1968.0
Crystal size/mm^3	0.06 × 0.02 × 0.01
Radiation	? (λ = 0.72932)
2Θ range for data collection/°	2.906 to 51.456
Index ranges	-11 ≤ h ≤ 11, -20 ≤ k ≤ 20, -32 ≤ l ≤ 32
Reflections collected	44533
Independent reflections	3930 [R_{int} = 0.0514, R_{sigma} = 0.0231]
Data/restraints/parameters	3930/12/340
Goodness-of-fit on F^2	1.077
Final R indexes [I>=2σ (I)]	R_1 = 0.0648, wR_2 = 0.1818
Final R indexes [all data]	R_1 = 0.0709, wR_2 = 0.1903
Largest diff. peak/hole / e Å^{-3}	0.41/-0.41

Table 2. Fractional Atomic Coordinates (×10⁴) and Equivalent Isotropic Displacement Parameters (Å²×10³) for aspidin BB (**1**). U_{eq} is defined as 1/3 of the trace of the orthogonalised U_{IJ} tensor.

Atom	*x*	*y*	*z*	U(eq)
O2	6799.1(15)	5103.7(8)	1929.0(5)	45.5(4)
O6'	7410.3(16)	5819.6(8)	2709.0(5)	46.8(4)
O2'	5344.6(17)	3616.8(10)	3451.5(6)	59.1(5)
O6	4612.2(18)	2932.7(10)	2642.2(6)	62.2(5)
O8	7306.3(19)	3839.4(10)	704.1(6)	64.1(5)
O4'	9137.0(19)	5059.8(11)	4181.0(6)	69.0(5)
O4	6109(2)	2765.1(10)	1084.9(7)	68.9(5)
O8'	6332(2)	3143.7(13)	4176.8(7)	82.5(6)
C6'	7315(2)	5321.2(12)	3072.2(7)	44.6(5)
C2	6343(2)	4416.8(11)	1900.6(7)	43.4(5)
C1'	6322(2)	4711.2(12)	3074.9(7)	45.4(5)
C1	5590(2)	4072.0(12)	2293.9(7)	45.7(5)
C5'	8247(2)	5463.9(13)	3447.0(7)	48.7(5)
C7	5228(2)	4613.7(12)	2695.5(7)	46.9(5)
C3	6542(2)	3955.0(12)	1475.7(8)	48.6(5)
C9	8054(3)	5019.2(12)	1057.9(8)	51.2(5)
C8	7286(2)	4254.2(13)	1069.9(8)	50.5(5)
C2'	6320(2)	4207.8(13)	3454.4(8)	51.1(5)
C6	5215(2)	3307.6(13)	2282.0(8)	50.9(6)
C5	5364(2)	2777.5(12)	1868.2(9)	54.0(6)
C12'	9236(2)	6154.9(14)	3429.7(8)	55.4(6)
C4'	8207(2)	4944.4(14)	3817.0(8)	54.9(6)
C4	6023(2)	3195.6(13)	1462.2(9)	54.3(6)
C3'	7272(2)	4289.3(14)	3832.3(8)	55.4(6)
C13	3870(2)	2481.4(14)	1726.3(10)	60.5(6)
C10	8915(3)	5147.4(14)	617.3(9)	60.5(6)
C12	6305(3)	2062.9(14)	1991.2(10)	65.3(7)
C8'	7236(3)	3682.6(18)	4197.3(9)	70.5(7)
C11	9727(4)	5914.4(16)	636.7(11)	80.6(9)
C7'	8640(4)	5630(2)	4512.1(10)	88.2(10)
C9'	8259(3)	3636(2)	4592.2(10)	85.6(9)
C10A	8134(6)	2812(3)	4831.5(18)	68.3(14)
C11A	9097(5)	2780(3)	5251.5(16)	82.6(16)
C10B	8018(10)	3204(7)	5048(3)	71(3)
C11B	8630(14)	2452(8)	4866(4)	76(4)

Table 3. Anisotropic Displacement Parameters (Å²×103) for aspidin BB (**1**). The Anisotropic displacement factor exponent takes the form: $-2\pi^2[h^2a^{*2}U_{11}+2hka^*b^*U_{12}+\ldots]$.

Atom	U_{11}	U_{22}	U_{33}	U_{23}	U_{13}	U_{12}
O2	45.6(8)	35.3(7)	55.7(9)	1.1(6)	-0.4(6)	0.8(6)
O6'	51.2(8)	37.2(7)	52.0(8)	1.5(6)	1.3(6)	-0.3(6)
O2'	56.5(9)	51.5(9)	69.3(10)	16.9(7)	5.5(7)	-1.7(8)
O6	59.7(10)	47.4(9)	79.4(12)	10.0(8)	2.8(8)	-12.1(8)
O8	69.0(11)	54.6(10)	68.5(10)	-15.0(8)	6.3(8)	-3.5(8)
O4'	66.6(11)	77.2(12)	63.2(10)	-0.9(8)	-18.3(8)	15.3(9)
O4	75.0(12)	49.6(9)	82.2(12)	-18.9(8)	3.9(9)	-9.0(8)
O8'	86.1(14)	78.3(13)	83.3(13)	34.8(11)	0.0(10)	3.9(11)

C6'	43.8(11)	39.0(11)	51.1(11)	0.0(8)	3.7(8)	9.4(9)
C2	37.7(10)	34.2(10)	58.2(12)	3.1(8)	-5.5(8)	2.4(8)
C1'	42.4(10)	41.7(11)	52.0(12)	2.0(8)	3.1(8)	6.5(9)
C1	38.6(10)	37.8(11)	60.7(12)	3.2(9)	-2.3(9)	1.1(9)
C5'	46.7(11)	44.5(11)	55.1(12)	-5.3(9)	-0.6(9)	9.6(10)
C7	40.1(11)	41.7(11)	59.0(12)	6.3(9)	2.9(9)	2.4(9)
C3	44.6(11)	40.1(11)	61.3(13)	-1.1(9)	-3.2(9)	0.9(9)
C9	56.0(12)	40.0(11)	57.7(12)	-1.0(9)	2.4(10)	6.1(10)
C8	48.6(12)	42.9(11)	60.0(13)	-5.8(9)	-1.6(9)	5.4(9)
C2'	47.0(12)	45.9(12)	60.4(13)	7.1(10)	5.5(9)	5.9(10)
C6	40.8(11)	41.4(11)	70.5(14)	7.9(10)	-3.7(9)	-3.9(9)
C5	47.4(12)	36.5(11)	78.1(15)	0.4(10)	-9.3(10)	-4.0(9)
C12'	52.0(12)	48.1(12)	66.0(14)	-10.1(10)	-7.5(10)	7.7(11)
C4'	50.5(12)	57.9(13)	56.1(13)	-1.8(10)	-5.1(10)	15.3(11)
C4	48.0(12)	43.6(12)	71.4(15)	-6.9(10)	-5.5(10)	0.1(10)
C3'	55.4(13)	54.0(13)	56.7(13)	8.7(10)	0.8(10)	11.7(11)
C13	49.9(13)	44.1(12)	87.5(17)	2.5(11)	-12.6(11)	-6.1(10)
C10	72.4(16)	47.3(12)	61.9(14)	-1.4(10)	10.2(12)	6.8(11)
C12	55.6(13)	41.8(12)	98.4(19)	0.8(12)	-14.7(13)	4.0(11)
C8'	70.9(17)	72.7(17)	67.9(16)	18.0(13)	-0.8(12)	19.1(15)
C11	107(2)	47.4(14)	88.0(19)	-1.6(13)	42.6(17)	-5.1(14)
C7'	101(2)	98(2)	64.9(17)	-18.5(15)	-28.5(16)	25.7(18)
C9'	83(2)	99(2)	74.4(18)	29.5(16)	-11.4(15)	18.4(18)
C10A	77(3)	64(3)	64(3)	14(3)	-12(2)	7(2)
C11A	77(3)	87(3)	84(3)	31(2)	-18(2)	0(2)
C10B	57(5)	104(8)	52(5)	-28(6)	1(4)	-10(5)
C11B	79(8)	84(9)	66(7)	18(6)	-8(6)	-2(6)

Table S4. Bond Lengths for aspidin BB **(1).**

Atom	Atom	Length/Å	Atom	Atom	Length/Å
O2	C2	1.248(2)	C5'	C4'	1.384(3)
O6'	C6'	1.348(2)	C3	C8	1.454(3)
O2'	C2'	1.363(3)	C3	C4	1.382(3)
O6	C6	1.343(3)	C9	C8	1.491(3)
O8	C8	1.267(3)	C9	C10	1.522(3)
O4'	C4'	1.381(3)	C2'	C3'	1.418(3)
O4'	C7'	1.438(4)	C6	C5	1.500(3)
O4	C4	1.312(3)	C5	C4	1.502(3)
O8'	C8'	1.254(4)	C5	C13	1.553(3)
C6'	C1'	1.399(3)	C5	C12	1.547(3)
C6'	C5'	1.413(3)	C4'	C3'	1.422(4)
C2	C1	1.459(3)	C3'	C8'	1.473(3)
C2	C3	1.465(3)	C10	C11	1.514(4)
C1'	C7	1.512(3)	C8'	C9'	1.493(4)
C1'	C2'	1.387(3)	C9'	C10A	1.566(5)
C1	C7	1.517(3)	C9'	C10B	1.520(9)
C1	C6	1.348(3)	C10A	C11A	1.513(6)
C5'	C12'	1.502(3)	C10B	C11B	1.499(13)

Table 5. Bond Angles for aspidin BB **(1)**.

Atom	Atom	Atom	Angle/°	Atom	Atom	Atom	Angle/°
C4'	O4'	C7'	112.97(19)	O6	C6	C1	123.3(2)
O6'	C6'	C1'	121.03(18)	O6	C6	C5	111.52(18)
O6'	C6'	C5'	116.21(19)	C1	C6	C5	125.1(2)
C1'	C6'	C5'	122.74(19)	C6	C5	C4	111.80(17)
O2	C2	C1	119.59(19)	C6	C5	C13	108.54(19)
O2	C2	C3	120.79(18)	C6	C5	C12	110.2(2)
C1	C2	C3	119.61(17)	C4	C5	C13	109.0(2)
C6'	C1'	C7	122.38(18)	C4	C5	C12	108.2(2)
C2'	C1'	C6'	117.6(2)	C12	C5	C13	109.09(18)
C2'	C1'	C7	119.93(19)	O4'	C4'	C5'	118.3(2)
C2	C1	C7	117.15(17)	O4'	C4'	C3'	118.9(2)
C6	C1	C2	119.7(2)	C5'	C4'	C3'	122.8(2)
C6	C1	C7	123.09(19)	O4	C4	C3	121.5(2)
C6'	C5'	C12'	119.8(2)	O4	C4	C5	113.87(19)
C4'	C5'	C6'	117.4(2)	C3	C4	C5	124.6(2)
C4'	C5'	C12'	122.8(2)	C2'	C3'	C4'	116.5(2)
C1'	C7	C1	117.52(17)	C2'	C3'	C8'	117.6(2)
C8	C3	C2	122.98(18)	C4'	C3'	C8'	125.8(2)
C4	C3	C2	118.6(2)	C11	C10	C9	111.3(2)
C4	C3	C8	118.4(2)	O8'	C8'	C3'	119.6(2)
C8	C9	C10	113.87(19)	O8'	C8'	C9'	115.9(2)
O8	C8	C3	118.6(2)	C3'	C8'	C9'	124.4(3)
O8	C8	C9	117.3(2)	C8'	C9'	C10A	109.5(3)
C3	C8	C9	124.00(19)	C8'	C9'	C10B	125.8(4)
O2'	C2'	C1'	116.8(2)	C11A	C10A	C9'	109.8(4)
O2'	C2'	C3'	120.4(2)	C11B	C10B	C9'	93.0(7)
C1'	C2'	C3'	122.8(2)				

Table S6. Torsion Angles for aspidin BB **(1)**.

A	B	C	D	Angle/°	A	B	C	D	Angle/°
O2	C2	C1	C7	8.2(3)	C5'	C6'	C1'	C7	173.47(18)
O2	C2	C1	C6	-173.68(19)	C5'	C6'	C1'	C2'	-3.4(3)
O2	C2	C3	C8	0.9(3)	C5'	C4'	C3'	C2'	-2.8(3)
O2	C2	C3	C4	179.70(19)	C5'	C4'	C3'	C8'	175.5(2)
O6'	C6'	C1'	C7	-5.1(3)	C7	C1'	C2'	O2'	3.0(3)
O6'	C6'	C1'	C2'	177.99(17)	C7	C1'	C2'	C3'	-177.0(2)
O6'	C6'	C5'	C12'	1.7(3)	C7	C1	C6	O6	-6.9(3)
O6'	C6'	C5'	C4'	-177.75(18)	C7	C1	C6	C5	170.94(19)
O2'	C2'	C3'	C4'	-177.0(2)	C3	C2	C1	C7	-171.28(17)
O2'	C2'	C3'	C8'	4.5(3)	C3	C2	C1	C6	6.8(3)
O6	C6	C5	C4	179.37(18)	C8	C3	C4	O4	-4.4(3)
O6	C6	C5	C13	59.1(2)	C8	C3	C4	C5	173.6(2)
O6	C6	C5	C12	-60.3(2)	C8	C9	C10	C11	-177.2(2)
O4'	C4'	C3'	C2'	178.7(2)	C2'	C1'	C7	C1	-89.6(2)
O4'	C4'	C3'	C8'	-3.0(4)	C2'	C3'	C8'	O8'	-3.6(4)
O8'	C8'	C9'	C10A	11.6(4)	C2'	C3'	C8'	C9'	173.6(3)
O8'	C8'	C9'	C10B	-23.2(7)	C6	C1	C7	C1'	94.0(2)
C6'	C1'	C7	C1	93.6(2)	C6	C5	C4	O4	-176.74(18)

C6'	C1'	C2'	O2'	179.98(18)	C6	C5	C4	C3	5.2(3)
C6'	C1'	C2'	C3'	0.0(3)	C12'	C5'	C4'	O4'	-1.3(3)
C6'	C5'	C4'	O4'	178.15(19)	C12'	C5'	C4'	C3'	-179.8(2)
C6'	C5'	C4'	C3'	-0.3(3)	C4'	C3'	C8'	O8'	178.1(2)
C2	C1	C7	C1'	-88.0(2)	C4'	C3'	C8'	C9'	-4.6(4)
C2	C1	C6	O6	175.08(19)	C4	C3	C8	O8	7.0(3)
C2	C1	C6	C5	-7.0(3)	C4	C3	C8	C9	-172.0(2)
C2	C3	C8	O8	-174.20(19)	C3'	C8'	C9'	C10A	-165.7(3)
C2	C3	C8	C9	6.9(3)	C3'	C8'	C9'	C10B	159.5(6)
C2	C3	C4	O4	176.7(2)	C13	C5	C4	O4	-56.8(3)
C2	C3	C4	C5	-5.3(3)	C13	C5	C4	C3	125.2(2)
C1'	C6'	C5'	C12'	-176.94(19)	C10	C9	C8	O8	-7.0(3)
C1'	C6'	C5'	C4'	3.6(3)	C10	C9	C8	C3	171.9(2)
C1'	C2'	C3'	C4'	3.0(3)	C12	C5	C4	O4	61.8(3)
C1'	C2'	C3'	C8'	-175.5(2)	C12	C5	C4	C3	-116.3(2)
C1	C2	C3	C8	-179.67(19)	C8'	C9'	C10A	C11A	-176.3(4)
C1	C2	C3	C4	-0.8(3)	C8'	C9'	C10B	C11B	89.8(7)
C1	C6	C5	C4	1.3(3)	C7'	O4'	C4'	C5'	83.1(3)
C1	C6	C5	C13	-119.0(2)	C7'	O4'	C4'	C3'	-98.3(3)
C1	C6	C5	C12	121.6(2)					

Table 7. Hydrogen Atom Coordinates (Å×10[4]) and Isotropic Displacement Parameters (Å[2]×10[3]) for aspidin BB **(1)**.

**Atom**	***x***	***y***	***z***	**U(eq)**
H6'	7060(30)	5555(8)	2424(6)	70
H2'	5460(20)	3290(13)	3737(8)	89
H6	4820(30)	3223(12)	2934(6)	93
H4	6610(30)	3072(10)	834(7)	103
H7A	5020.44	5140.45	2566.01	56
H7B	4345.21	4418.43	2840.81	56
H9A	8697.41	5046.77	1329.11	61
H9B	7357.64	5451.06	1088.07	61
H12A	9614(16)	6210(6)	3116(5)	83
H12B	10013(16)	6072(5)	3647(6)	83
H12C	8724(9)	6631(8)	3515(6)	83
H13A	3448(11)	2160(10)	1991(5)	91
H13B	3949(4)	2139(10)	1437(6)	91
H13C	3233(13)	2951(8)	1658(6)	91
H10A	9588.48	4706.55	577.24	73
H10B	8272.37	5152.7	345.45	73
H12D	7269(19)	2249(4)	2080(7)	98
H12E	6376(17)	1708(9)	1716(6)	98
H12F	5876(14)	1771(9)	2257(7)	98
H11A	10250(20)	5999(7)	329(7)	121
H11B	10440(20)	5894(6)	903(7)	121
H11C	9036(14)	6369(9)	691(8)	121
H7'A	8580(30)	6156(12)	4360(4)	132
H7'B	9310(20)	5658(11)	4779(8)	132
H7'C	7680(20)	5471(9)	4628(7)	132
H9'A	8050.3	4055.41	4820.78	103
H9'B	9235.17	3713.92	4476	103

H9'C	8476.06	4186.52	4677.96	103
H9'D	9141.53	3415.31	4460.38	103
H10C	7142.4	2719.37	4928.67	82
H10D	8403.24	2394.64	4608.35	82
H11D	10140(30)	2860(20)	5146(4)	124
H11E	9000(30)	2235(19)	5413(9)	124
H11F	8820(20)	3223(19)	5484(9)	124
H10E	8566.54	3427.53	5309.78	85
H10F	7004.62	3159.36	5132.01	85
H11G	7970(60)	2240(30)	4550(30)	114
H11H	8600(70)	1980(30)	5150(20)	114
H11I	9790(80)	2556(14)	4750(20)	114

Table 8. Atomic Occupancy for aspidin BB **(1).**

**Atom**	***Occupancy***	**Atom**	***Occupancy***	**Atom**	***Occupancy***
H9'A	0.710(9)	H9'B	0.710(9)	H9'C	0.290(9)
H9'D	0.290(9)	C10A	0.710(9)	H10C	0.710(9)
H10D	0.710(9)	C11A	0.710(9)	H11D	0.710(9)
H11E	0.710(9)	H11F	0.710(9)	C10B	0.290(9)
H10E	0.290(9)	H10F	0.290(9)	C11B	0.290(9)
H11G	0.290(9)	H11H	0.290(9)	H11I	0.290(9)

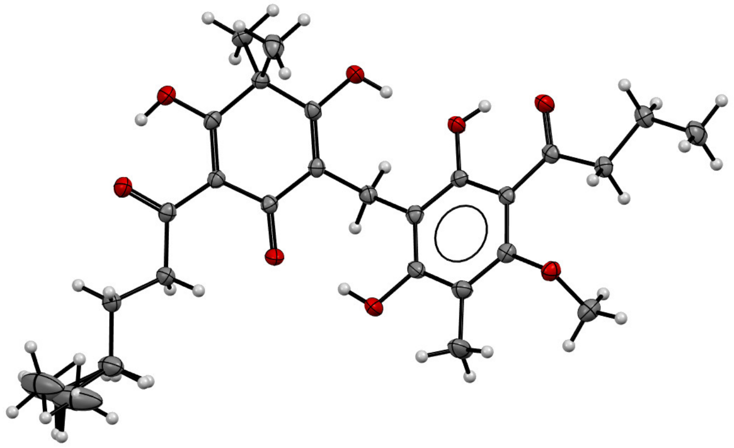

Table 9. X-ray crystal data and structure refinement of aspidin VB **(2)**.

**Identification code**	**compound 2**
Empirical formula	$C_{27}H_{36}O_8$
Formula weight	488.56
Temperature/K	150.01(10)
Crystal system	monoclinic
Space group	$P2_1/c$
a/Å	9.1088(5)
b/Å	16.5818(10)
c/Å	16.7309(9)
α/°	90
β/°	91.289(5)
γ/°	90
Volume/Å^3	2526.4(2)
Z	4
ρ_{calc}g/cm^3	1.284
μ/mm^{-1}	0.094
F(000)	1048.0
Crystal size/mm^3	0.100 × 0.050 × 0.50
Radiation	Mo Kα (λ = 0.71073)
2Θ range for data collection/°	8.184 to 51.362
Index ranges	-9 ≤ h ≤ 11, -20 ≤ k ≤ 20, -20 ≤ l ≤ 20
Reflections collected	1482 »
Independent reflections	4697 [R_{int} = 0.0425, R_{sigma} = 0.0477]
Data/restraints/parameters	4697/63/346
Goodness-of-fit on F^2	1.048
Final R indexes [I>=2σ (I)]	R_1 = 0.0438, wR_2 = 0.1132
Final R indexes [all data]	R_1 = 0.0583, wR_2 = 0.1212
Largest diff. peak/hole / e Å^{-3}	0.38/-0.19

Table 10. Fractional Atomic Coordinates (×10[4]) and Equivalent Isotropic Displacement Parameters ($Å^2×10^3$) for aspidin VB **(2)**. U_{eq} is defined as 1/3 of the trace of the orthogonalised U_{IJ} tensor.

Atom	*x*	*y*	*z*	U(eq)
O2'	4852.9(11)	2950.5(7)	1316.8(6)	22.6(3)
O4'	8934.0(11)	4287.5(7)	111.0(6)	22.8(3)
O2	8066.7(12)	2901.4(8)	4000.1(7)	27.4(3)
O8'	4500.0(12)	3672.6(8)	54.6(7)	27.8(3)
O6	3424.6(12)	2907.1(8)	2699.6(7)	28.4(3)
O8	6229.0(12)	3841.7(7)	6062.0(7)	25.2(3)
O6'	9497.9(12)	3059.1(8)	2645.1(7)	28.7(3)
O4	3884.4(12)	3763.0(8)	5368.0(7)	29.8(3)
C8'	5826.5(16)	3852.6(9)	9.4(9)	19.9(3)
C2'	6279.6(16)	3189.9(9)	1323.6(9)	18.0(3)
C3'	6841.9(16)	3630.4(9)	668.9(9)	18.4(3)
C4'	8352.9(17)	3839.8(9)	720.3(9)	20.1(3)
C1	5791.9(16)	2855.3(9)	3325.9(9)	19.0(3)
C2	6720.6(16)	3036.4(9)	4023.0(9)	19.3(3)
C6	4342.8(17)	3026.8(10)	3327.4(9)	20.4(3)
C7	6498.5(17)	2433.7(10)	2629.5(9)	20.4(3)
C1'	7140.6(16)	2966.0(9)	1986.3(9)	19.5(3)
C5	3530.9(16)	3350.9(10)	4031.2(9)	20.6(3)
C3	6056.2(16)	3362.2(9)	4743.9(9)	19.0(3)
C9	8537.3(17)	3496.3(11)	5541.9(9)	24.0(4)
C8	6897.4(17)	3575.1(9)	5464.5(9)	20.3(3)
C4	4545.3(17)	3489.1(10)	4743.6(9)	20.5(3)
C6'	8601.6(17)	3219.4(10)	2011.5(9)	21.7(3)
C5'	9233.9(17)	3654.3(10)	1381.0(10)	24.4(4)
C9'	6290.1(17)	4296.5(10)	-726.9(9)	22.7(3)
C10	9191.9(17)	3827.3(11)	6322.8(10)	25.6(4)
C15	2333.1(17)	2737.3(11)	4254.8(10)	25.9(4)
C10'	5051.7(18)	4431.0(10)	-1342.7(10)	25.7(4)
C14	2799(2)	4162.3(11)	3815.8(11)	31.7(4)
C7'	9935.0(19)	3858.1(11)	-385.9(11)	29.9(4)
C12'	10802.2(19)	3939.1(13)	1462.8(12)	36.9(5)
C11'	5606(2)	4889.4(12)	-2063.9(11)	34.4(4)
C11	10834.1(19)	3656.2(13)	6392.3(12)	36.9(5)
C12A	11647(13)	4089(8)	7070(6)	57(3)
C13A	11808(9)	4958(5)	6896(10)	89(3)
C12B	11500(20)	3955(12)	7188(11)	59(3)
C13B	11430(16)	4767(11)	7358(13)	90(5)

Table 11. Anisotropic Displacement Parameters (Å²×103) for aspidin VB **(2)**. The Anisotropic displacement factor exponent takes the form: $-2\pi^2[h^2a^{*2}U_{11}+2hka^*b^*U_{12}+…]$.

Atom	U_{11}	U_{22}	U_{33}	U_{23}	U_{13}	U_{12}
O2'	17.9(5)	31.2(7)	18.4(5)	2.2(5)	-2.2(4)	-4.5(5)
O4'	22.1(5)	23.4(6)	23.2(6)	4.5(5)	3.6(4)	-1.8(4)
O2	18.6(6)	42.7(8)	20.7(6)	-3.4(5)	-1.3(4)	8.0(5)
O8'	20.5(6)	39.3(7)	23.3(6)	5.0(5)	-4.0(5)	-3.6(5)
O6	18.8(6)	48.3(8)	17.9(6)	-1.8(5)	-1.6(4)	-2.4(5)
O8	23.6(6)	31.5(7)	20.5(6)	-5.0(5)	2.6(5)	-0.4(5)

O6'	19.8(6)	45.9(8)	20.2(6)	6.3(5)	-2.8(4)	2.3(5)
O4	21.1(6)	47.8(8)	20.6(6)	-5.3(5)	2.9(5)	2.8(5)
C8'	21.8(8)	18.4(8)	19.3(8)	-4.4(6)	-1.4(6)	0.6(6)
C2'	16.7(7)	17.7(8)	19.5(7)	-4.3(6)	1.0(6)	-1.2(6)
C3'	20.2(7)	17.4(8)	17.5(7)	-2.8(6)	-0.4(6)	1.8(6)
C4'	21.9(8)	17.6(8)	20.7(8)	-0.2(6)	1.9(6)	-0.5(6)
C1	21.8(8)	18.1(8)	17.0(7)	1.9(6)	1.2(6)	-0.7(6)
C2	20.2(7)	19.3(8)	18.4(7)	2.9(6)	1.1(6)	1.2(6)
C6	21.9(8)	22.0(8)	17.4(7)	3.5(6)	-1.4(6)	-3.7(6)
C7	21.6(8)	21.1(8)	18.5(8)	-0.8(6)	-0.4(6)	1.0(6)
C1'	21.3(8)	19.0(8)	18.4(7)	-1.7(6)	1.3(6)	1.3(6)
C5	17.6(7)	24.4(9)	19.8(8)	3.8(6)	1.1(6)	1.6(6)
C3	19.6(7)	19.5(8)	18.0(7)	0.9(6)	1.7(6)	-0.2(6)
C9	19.7(8)	30.6(9)	21.5(8)	-3.0(7)	-0.4(6)	-0.6(6)
C8	22.0(8)	19.0(8)	20.0(8)	1.5(6)	2.1(6)	-2.0(6)
C4	21.3(7)	21.3(8)	19.2(8)	2.6(6)	4.0(6)	-1.6(6)
C6'	19.4(7)	26.3(9)	19.3(8)	-1.4(6)	-3.1(6)	3.4(6)
C5'	19.0(8)	27.8(9)	26.2(9)	1.2(7)	-0.9(6)	-1.8(6)
C9'	25.1(8)	22.8(8)	20.1(8)	1.3(6)	-0.5(6)	0.6(6)
C10	22.8(8)	31.6(9)	22.3(8)	-4.8(7)	0.2(6)	-0.5(7)
C15	18.0(8)	33.6(10)	26.1(8)	3.3(7)	2.5(6)	-3.7(7)
C10'	28.5(8)	26.6(9)	21.8(8)	2.1(7)	-3.1(7)	0.6(7)
C14	38.8(10)	31.0(10)	25.3(9)	4.5(7)	-0.2(7)	8.6(8)
C7'	28.5(9)	31.4(10)	30.1(9)	-0.2(7)	7.9(7)	1.0(7)
C12'	22.6(9)	53.2(13)	34.7(10)	11.8(9)	-6.2(7)	-10.1(8)
C11'	37.6(10)	39.1(11)	26.0(9)	9.7(8)	-5.4(8)	-1.2(8)
C11	25.6(9)	44.8(12)	40.0(11)	-13.0(9)	-4.2(8)	2.3(8)
C12A	29(3)	101(6)	41(3)	-47(4)	-1(2)	-5(3)
C13A	41(3)	66(4)	157(9)	-58(5)	-29(4)	11(3)
C12B	25(5)	71(5)	79(7)	1(4)	-16(4)	7(4)
C13B	48(6)	108(8)	112(10)	-72(8)	-27(6)	13(6)

Table S12. Bond Lengths for aspidin VB **(2)**.

Atom	Atom	Length/Å	Atom	Atom	Length/Å
O2'	C2'	1.3587(18)	C6	C5	1.504(2)
O4'	C4'	1.3765(19)	C7	C1'	1.519(2)
O4'	C7'	1.437(2)	C1'	C6'	1.395(2)
O2	C2	1.2479(18)	C5	C4	1.509(2)
O8'	C8'	1.2485(19)	C5	C14	1.541(2)
O6	C6	1.3427(18)	C5	C15	1.544(2)
O8	C8	1.2621(19)	C3	C4	1.392(2)
O6'	C6'	1.3498(18)	C3	C8	1.457(2)
O4	C4	1.299(2)	C9	C8	1.502(2)
C8'	C3'	1.471(2)	C9	C10	1.526(2)
C8'	C9'	1.504(2)	C6'	C5'	1.411(2)
C2'	C1'	1.394(2)	C5'	C12'	1.508(2)
C2'	C3'	1.422(2)	C9'	C10'	1.527(2)
C3'	C4'	1.420(2)	C10	C11	1.524(2)
C4'	C5'	1.386(2)	C10'	C11'	1.522(2)
C1	C6	1.350(2)	C11	C12A	1.520(9)

C1	C2	1.457(2)	C11	C12B	1.533(17)
C1	C7	1.515(2)	C12A	C13A	1.478(13)
C2	C3	1.465(2)	C12B	C13B	1.38(2)

Table 13. Bond Angles for aspidin VB **(2)**.

Atom	Atom	Atom	Angle/°	Atom	Atom	Atom	Angle/°
C4'	O4'	C7'	114.89(13)	C4	C5	C14	107.83(13)
O8'	C8'	C3'	119.20(14)	C6	C5	C15	108.47(13)
O8'	C8'	C9'	117.17(13)	C4	C5	C15	109.40(12)
C3'	C8'	C9'	123.63(13)	C14	C5	C15	109.12(13)
O2'	C2'	C1'	116.73(13)	C4	C3	C8	117.71(14)
O2'	C2'	C3'	120.32(13)	C4	C3	C2	118.83(13)
C1'	C2'	C3'	122.94(13)	C8	C3	C2	123.46(13)
C4'	C3'	C2'	116.39(13)	C8	C9	C10	114.27(13)
C4'	C3'	C8'	125.20(14)	O8	C8	C3	119.09(14)
C2'	C3'	C8'	118.39(13)	O8	C8	C9	117.30(13)
O4'	C4'	C5'	119.01(13)	C3	C8	C9	123.61(13)
O4'	C4'	C3'	118.38(13)	O4	C4	C3	121.90(14)
C5'	C4'	C3'	122.48(14)	O4	C4	C5	113.68(13)
C6	C1	C2	120.34(14)	C3	C4	C5	124.40(14)
C6	C1	C7	122.08(13)	O6'	C6'	C1'	121.60(14)
C2	C1	C7	117.51(13)	O6'	C6'	C5'	115.97(13)
O2	C2	C1	119.46(14)	C1'	C6'	C5'	122.43(14)
O2	C2	C3	121.05(13)	C4'	C5'	C6'	118.09(14)
C1	C2	C3	119.48(13)	C4'	C5'	C12'	122.22(15)
O6	C6	C1	123.99(14)	C6'	C5'	C12'	119.60(14)
O6	C6	C5	110.90(13)	C8'	C9'	C10'	114.05(13)
C1	C6	C5	125.09(13)	C11	C10	C9	111.23(14)
C1	C7	C1'	116.98(13)	C11'	C10'	C9'	110.79(14)
C2'	C1'	C6'	117.58(14)	C12A	C11	C10	115.4(4)
C2'	C1'	C7	119.97(13)	C10	C11	C12B	112.1(7)
C6'	C1'	C7	122.39(13)	C13A	C12A	C11	111.3(8)
C6	C5	C4	111.69(12)	C13B	C12B	C11	118.4(13)
C6	C5	C14	110.30(13)				

Table S14. Torsion Angles for aspidin VB **(2)**.

A	B	C	D	Angle/°	A	B	C	D	Angle/°
O2'	C2'	C3'	C4'	-178.66(13)	O2	C2	C3	C8	2.1(2)
C1'	C2'	C3'	C4'	-0.2(2)	C1	C2	C3	C8	-178.87(14)
O2'	C2'	C3'	C8'	3.0(2)	C4	C3	C8	O8	2.0(2)
C1'	C2'	C3'	C8'	-178.49(14)	C2	C3	C8	O8	-179.02(14)
O8'	C8'	C3'	C4'	-175.87(15)	C4	C3	C8	C9	-177.99(14)
C9'	C8'	C3'	C4'	3.6(2)	C2	C3	C8	C9	1.0(2)
O8'	C8'	C3'	C2'	2.3(2)	C10	C9	C8	O8	-5.3(2)
C9'	C8'	C3'	C2'	-178.19(14)	C10	C9	C8	C3	174.67(15)
C7'	O4'	C4'	C5'	74.18(19)	C8	C3	C4	O4	-2.2(2)
C7'	O4'	C4'	C3'	-109.83(16)	C2	C3	C4	O4	178.76(15)
C2'	C3'	C4'	O4'	-178.07(13)	C8	C3	C4	C5	175.99(14)
C8'	C3'	C4'	O4'	0.1(2)	C2	C3	C4	C5	-3.1(2)
C2'	C3'	C4'	C5'	-2.2(2)	C6	C5	C4	O4	-179.44(13)

C8'	C3'	C4'	C5'	175.98(15)	C14	C5	C4	O4	59.22(18)
C6	C1	C2	O2	-177.35(15)	C15	C5	C4	O4	-59.34(18)
C7	C1	C2	O2	5.7(2)	C6	C5	C4	C3	2.3(2)
C6	C1	C2	C3	3.6(2)	C14	C5	C4	C3	-119.08(17)
C7	C1	C2	C3	-173.33(14)	C15	C5	C4	C3	122.36(17)
C2	C1	C6	O6	177.39(14)	C2'	C1'	C6'	O6'	177.21(14)
C7	C1	C6	O6	-5.9(2)	C7	C1'	C6'	O6'	-5.5(2)
C2	C1	C6	C5	-4.6(2)	C2'	C1'	C6'	C5'	-3.0(2)
C7	C1	C6	C5	172.20(14)	C7	C1'	C6'	C5'	174.31(15)
C6	C1	C7	C1'	92.28(18)	O4'	C4'	C5'	C6'	177.78(14)
C2	C1	C7	C1'	-90.87(17)	C3'	C4'	C5'	C6'	2.0(2)
O2'	C2'	C1'	C6'	-178.80(14)	O4'	C4'	C5'	C12'	1.1(3)
C3'	C2'	C1'	C6'	2.7(2)	C3'	C4'	C5'	C12'	-174.69(16)
O2'	C2'	C1'	C7	3.9(2)	O6'	C6'	C5'	C4'	-179.43(15)
C3'	C2'	C1'	C7	-174.68(14)	C1'	C6'	C5'	C4'	0.7(3)
C1	C7	C1'	C2'	-91.56(17)	O6'	C6'	C5'	C12'	-2.7(2)
C1	C7	C1'	C6'	91.23(18)	C1'	C6'	C5'	C12'	177.48(17)
O6	C6	C5	C4	179.97(13)	O8'	C8'	C9'	C10'	-2.7(2)
C1	C6	C5	C4	1.7(2)	C3'	C8'	C9'	C10'	177.79(14)
O6	C6	C5	C14	-60.13(17)	C8	C9	C10	C11	174.69(15)
C1	C6	C5	C14	121.59(17)	C8'	C9'	C10'	C11'	179.48(14)
O6	C6	C5	C15	59.32(16)	C9	C10	C11	C12A	169.3(6)
C1	C6	C5	C15	-118.95(17)	C9	C10	C11	C12B	-177.8(9)
O2	C2	C3	C4	-178.91(15)	C10	C11	C12A	C13A	-72.7(8)
C1	C2	C3	C4	0.1(2)	C10	C11	C12B	C13B	-61.0(16)

Table 15. Hydrogen Atom Coordinates (Å×10^4) and Isotropic Displacement Parameters (Å^2×10^3) for aspidin VB **(2)**. **.**

**Atom**	***x***	***y***	***z***	**U(eq)**
H2'	4422.82	3127.1	903.32	34
H6	3910.8	2868.65	2280.6	43
H6'	8991.22	2961.8	3047.22	43
H4	4498.15	3822.47	5745.65	45
H7A	5753.41	2077.92	2372.67	25
H7B	7292.82	2083.18	2845.51	25
H9A	8987.29	3783.41	5090	29
H9B	8801.37	2919.15	5497.9	29
H9'A	7084.71	3988.42	-981.42	27
H9'B	6697.28	4826.96	-565.03	27
H10A	9025.12	4416.75	6346.48	31
H10B	8690.57	3577.1	6779.49	31
H15A	1640.41	2670.84	3802.89	39
H15B	1807.29	2934.52	4720.56	39
H15C	2791.27	2216.97	4382.84	39
H10C	4649.51	3903.64	-1517.87	31
H10D	4250.88	4740.25	-1095.2	31
H14A	3554.35	4554.66	3674.48	48
H14B	2258.43	4361.72	4275.71	48
H14C	2117.2	4086.68	3360.36	48
H7'A	10223.41	4204.22	-830.41	45

H7'B	9455.29	3371.39	-597.28	45
H7'C	10809.37	3705.68	-68.87	45
H12A	11467.27	3489.48	1354.67	55
H12B	10988.06	4138.27	2007.15	55
H12C	10970.57	4374.56	1079.58	55
H11A	4793.43	4973.37	-2448.97	52
H11B	6382.34	4577.17	-2316.37	52
H11C	5998.04	5412.9	-1890.83	52
H11D	10973.98	3068.27	6463.68	44
H11E	11289.26	3808.63	5882.23	44
H11F	11336.94	3923.76	5945.85	44
H11G	11000.29	3068.22	6345.37	44
H12D	11101.19	4020.86	7570.44	68
H12E	12630.75	3844.72	7149.73	68
H13A	12377.46	5026.86	6410.57	133
H13B	12318.75	5222.95	7345.87	133
H13C	10834.58	5200.62	6815.97	133
H12F	11014.48	3661.31	7623.25	70
H12G	12549.69	3795.85	7205.98	70
H13D	11726.1	5077.59	6889.78	135
H13E	12090.85	4890.76	7811.42	135
H13F	10421.47	4910.01	7492.75	135

Table S16. Atomic Occupancy for aspidin VB **(2)**.

**Atom**	***Occupancy***	**Atom**	***Occupancy***	**Atom**	***Occupancy***
H11D	0.60(2)	H11E	0.60(2)	H11F	0.40(2)
H11G	0.40(2)	C12A	0.60(2)	H12D	0.60(2)
H12E	0.60(2)	C13A	0.60(2)	H13A	0.60(2)
H13B	0.60(2)	H13C	0.60(2)	C12B	0.40(2)
H12F	0.40(2)	H12G	0.40(2)	C13B	0.40(2)
H13D	0.40(2)	H13E	0.40(2)	H13F	0.40(2)
